# Association of Structured Preclinical Phantom Training and OSCE/OSPE-Based Assessment with Endodontic Procedural Complications Among Dental Students: A Retrospective Cohort Study

**DOI:** 10.3390/diagnostics16193113

**Published:** 2026-09-24

**Authors:** Esma Dinger, Özlem Sivas Yılmaz, Melis Oya Ateş, Münevver Ayşegül Görgülü Pınar, Ayşe Nur Atay Altunkaya, Gül Sena Eser

**Affiliations:** Department of Endodontics, Faculty of Dentistry, Bolu Abant İzzet Baysal University, 14030 Bolu, Turkey; ozlem.sivasyilmaz@ibu.edu.tr (Ö.S.Y.); melisoyaates@hotmail.com (M.O.A.); munevver_gorgulu05@hotmail.com (M.A.G.P.); aysenurraty@gmail.com (A.N.A.A.); gl.senaeser@gmail.com (G.S.E.)

**Keywords:** objective structured clinical examination (OSCE), objective structured practical examination (OSPE), phantom laboratory, procedural complications, root canal treatment

## Abstract

**Background/Objectives:** The aim of this retrospective cohort study was to evaluate the association between structured phantom laboratory training and Objective Structured Clinical Examination (OSCE)/Objective Structured Practical Examination (OSPE)-based assessment and procedural complications observed during root canal treatments performed by undergraduate dental students in the clinical setting. **Methods:** Periapical radiographs obtained from 1204 completed root canal treatments performed at the Department of Endodontics, Faculty of Dentistry, Bolu Abant İzzet Baysal University, were evaluated. The teeth that underwent root canal treatment in the clinic by students who had not received structured phantom laboratory training and OSCE/OSPE-based assessment and by those who had received this training were divided into two groups, comprising 722 teeth and 482 teeth, respectively. The association between training status and overall procedural complications was analyzed using generalized linear mixed models (GLMMs), taking into account the clustering of treatments within students. **Results:** The prevalence of at least one procedural complication was 42.9% in the non-training group and 37.8% in the training group. In the primary GLMM analysis adjusted for the number of treated teeth per student, training status was not significantly associated with the overall occurrence of procedural complications (odds ratio (OR) = 0.58, 95% confidence interval (CI): 0.29–1.16, *p* = 0.069). In the secondary/exploratory analyses, lower odds of canal transportation (OR = 0.12, 95% CI: 0.02–0.94, *p* = 0.044) and overfilling (OR = 0.56, 95% CI: 0.38–0.82, *p* = 0.003) were observed in the training group, whereas the odds of missed canals were higher (OR = 6.23, 95% CI: 2.08–18.65, *p* = 0.001). The likelihood of procedural complications was higher in molar teeth than in anterior teeth (OR = 5.84, 95% CI: 3.66–9.46, *p* < 0.001). **Conclusions:** Although structured phantom laboratory training and OSCE/OSPE-based assessment were not significantly associated with the overall occurrence of procedural complications, exposure to the revised educational program was associated with lower odds of certain technical errors.

## 1. Introduction

Root canal treatment is one of the most commonly performed procedures in clinical endodontic practice. The primary objectives of root canal treatment are to remove infected tissues from the root canal system through adequate canal preparation and irrigation, disinfect the canal system, and subsequently obturate it with a hermetic seal to prevent reinfection [1]. When root canal treatment is performed in accordance with established endodontic principles and adapted to the root canal anatomy, high success rates have been reported [2]. However, these success rates may vary depending on the clinician’s clinical experience and level of expertise. Previous studies have reported that the success rates of root canal treatments performed by undergraduate dental students range from 33% to 70% [3,4]. This considerable variation in treatment outcomes is largely attributable to the numerous procedural complications encountered during clinical endodontic training in undergraduate dental education [5].

Procedural complications encountered during endodontic treatment are closely associated with students’ level of technical knowledge and manual skills and may occur at any stage of treatment, including diagnosis, treatment planning, administration of local anesthesia, access cavity preparation, canal instrumentation, irrigation, and obturation [6,7,8]. The outcome of root canal treatment is primarily evaluated by radiographic analysis in conjunction with clinical findings, as both are important determinants of treatment success and prognosis [9,10]. Radiographic assessment may reveal procedural complications known to compromise treatment outcomes, including inadequate canal preparation, ledge formation, canal transportation, instrument separation, perforation, and underfilling or overfilling of the root canal system [8].

Accordingly, undergraduate dental students are expected to acquire sufficient experience to perform root canal treatment without procedural complications. The European Society of Endodontology (ESE) states in its undergraduate curriculum guidelines that dental students are required to acquire the competencies necessary to perform successful root canal treatment during their undergraduate education [11].

Contemporary dental education is no longer focused solely on the delivery of theoretical knowledge but also emphasizes the systematic development of cognitive, psychomotor, and affective skills [12]. Although expert curriculum guidelines define the scope of undergraduate endodontic education, the number of clinical cases required to achieve competency, as well as the optimal preclinical and clinical teaching methodologies, remain subjects of ongoing debate [13,14]. Previous studies have demonstrated that limited preclinical and clinical training may hinder the attainment of competence and successful outcomes in root canal treatment [15,16].

In this context, structured educational approaches, including preclinical training in phantom laboratories and structured assessment methods such as the Objective Structured Clinical Examination (OSCE) and Objective Structured Practical Examination (OSPE), have become increasingly established as reliable tools for improving and assessing students’ clinical competence over time [17,18]. There is growing evidence in the literature that structured assessment methods, such as OSCE/OSPE, together with phantom laboratory training, enhance both students’ self-perceived competence and their clinical performance. Furthermore, these educational approaches provide greater standardization and more objective assessment than traditional teaching methods [19,20].

Previous studies have investigated the effects of various modifications to preclinical endodontic education [21,22,23]. However, studies evaluating the impact of OSCE/OSPE-based assessment and phantom laboratory training on the quality of root canal treatments performed on patients during undergraduate clinical practice, as well as their association with procedural complications, remain limited. To our knowledge, this is the first retrospective cohort study to evaluate whether exposure to an educational program incorporating structured phantom laboratory training together with OSCE/OSPE-based assessment is associated with procedural complications observed during undergraduate clinical endodontic treatment.

The aim of this retrospective cohort study was to evaluate the association between exposure to an educational program incorporating structured phantom laboratory training and OSCE/OSPE-based assessment and procedural complications observed during root canal treatments performed by undergraduate dental students in the clinical setting.

The null hypothesis of this study was formulated as follows: “There is no difference in the frequency of procedural complications observed during root canal treatments performed in the clinical setting between students who received structured phantom laboratory training and OSCE/OSPE-based assessment and students who were not included in this training and assessment process.”

## 2. Materials and Methods

This retrospective study was conducted in accordance with the ethical standards of the 2024 Declaration of Helsinki and was approved by the Bolu Abant İzzet Baysal University Non-Interventional Research Ethics Committee (7 April 2026, No:2026/127). In addition, written informed consent was obtained from all patients for the use of their periapical radiographs for research purposes. This study was reported in accordance with the Strengthening the Reporting of Observational Studies in Epidemiology (STROBE) guidelines.

In this study, root canal treatments performed during the endodontic clinical practice courses by fourth-year dental students who began treating patients during the 2023–2024 academic year at the Department of Endodontics, Faculty of Dentistry, Bolu Abant İzzet Baysal University, who had not received phantom laboratory training and whose treatment procedures and patient management had not been evaluated using OSCE/OSPE-based assessment, were retrospectively compared, in terms of procedural complications and errors, with root canal treatments performed during the endodontic clinical practice courses by fourth-year dental students who began treating patients during the 2024–2025 academic year, who had received phantom laboratory training and whose treatment procedures and patient management had been evaluated using OSCE/OSPE-based assessment.

The cases included in this study consisted of completed root canal treatments performed on teeth diagnosed as requiring root canal treatment at the Department of Oral Diagnosis and Radiology, Faculty of Dentistry, Bolu Abant İzzet Baysal University. Cases with radiographic images of insufficient quality and cases treated by students who were repeating the academic year were excluded from the study.

Available treatment-stage radiographs obtained from the digital radiographic records of 1204 treated teeth were evaluated. All eligible treatments performed during the predefined study periods were included; therefore, no a priori sample size calculation was performed.

The evaluations were performed on radiographs recorded in the Metasoft DentAssist software (version 4.1.220, Metasoft Bilgi Teknolojileri A.Ş., Eskişehir, Türkiye), which is used at the Faculty of Dentistry, Bolu Abant İzzet Baysal University, by three endodontists with at least 5 years of clinical experience who were blinded to the group allocation during the assessment.

Interexaminer agreement among the three blinded endodontists was assessed using Fleiss’ kappa coefficient, as the evaluated procedural complications were categorical variables. In cases of disagreement, the final classification was determined by majority agreement among the three evaluators.

### 2.1. Data Groups Evaluated

Group 1 (Control Group): Periapical radiographs of 722 root canal treatment cases performed on patients in the clinic by fourth-year 90 dental students during the 2023–2024 academic year who had not received phantom laboratory training and had not undergone OSCE/OSPE-based assessment (non-training group).

Group 2: Periapical radiographs of 482 root canal treatment cases performed on patients in the clinic by fourth-year 112 dental students during the 2024–2025 academic year who had received phantom laboratory training and had undergone OSCE/OSPE-based assessment (structured training/OSCE/OSPE group or training group).

During the study period, the teaching staff, student-to-faculty ratio, file system used, irrigation protocol, obturation technique and material (gutta-percha, root canal sealer), case selection criteria, and radiographic quality remained unchanged. However, the number of patients treated by the students in the clinic varied according to the patient volume during the respective academic years.

### 2.2. Educational Background of the Student Groups

The fourth-year students in the 2023–2024 academic year had received traditional preclinical endodontic education during the previous academic year. The Endodontic Practice II course was delivered throughout the academic year for 4 h per week, and students performed manual root canal treatment on extracted teeth in the preclinical laboratory. Phantom laboratory training using simulated models was not included in this course. During the second semester, students also attended a Clinical Observation course for 2 h per week. This course included clinical observation across all dental disciplines; therefore, the endodontic component consisted of a two-week observation period in the endodontic clinic. Scenario-based structured OSCE/OSPE assessment was not incorporated into the Clinical Observation course for this cohort.

The fourth-year students in the 2024–2025 academic year received preclinical endodontic education during the first semester. In contrast to the previous cohort, phantom laboratory training was incorporated into the preclinical course, in which students performed root canal treatment using both simulated teeth and extracted natural teeth embedded in phantom models to reproduce a clinical setting. During the second semester, students attended a Clinical Observation course for 16 h per week. As in the previous cohort, this course included clinical observation across all dental disciplines, and the endodontic component consisted of a two-week observation period in the endodontic clinic. In this cohort, however, scenario-based structured OSCE/OSPE assessment was incorporated into the Clinical Observation course. All structured practical assessments were performed by calibrated instructors using standardized checklists.

### 2.3. Assessment Criteria for Procedural Complications

Each case was evaluated for the following procedural complications. The operational definitions of each complication are presented below [8,23].

- Presence of instrument separation: Presence of a separated instrument within the root canal.
- Ledge formation: The instrument is unable to reach its working length by following its original canal path due to an artificial irregularity created in the canal wall.
- Canal transportation: A deviation of the prepared canal from its original canal path, identified radiographically by comparing the canal trajectory across the available treatment-stage radiographs, particularly in the apical third.
- Lateral/Furcal perforation: A radiographically identified communication between the root canal system and the external root surface, occurring laterally or in the furcation region.
- Inadequate preparation: Inadequate canal enlargement, identified radiographically by a thin root canal filling.
- Overfilling: Presence of gutta-percha extending beyond the apical foramen.
- Underfilling: Root canal filling terminating >2 mm short of the radiographic apex.
- Inadequate homogeneity of the root canal filling: Non-uniform radiopacity of the root canal filling with the presence of voids and radiolucent areas.
- Missed canal(s): A radiographically identified canal that remained unfilled (e.g., second mesiobuccal canal).

Complication categories were not mutually exclusive; therefore, a single tooth could be classified as having more than one procedural complication when the predefined radiographic criteria for multiple outcomes were simultaneously met. Inadequate preparation was distinguished from underfilling and poor homogeneity based on the radiographic appearance of insufficient canal enlargement, whereas underfilling was defined according to the apical termination of the root canal filling and poor homogeneity according to the presence of voids or radiolucent areas within the filling.

Because of the retrospective design of the study, available periapical radiographs obtained at different stages of treatment, including working-length radiographs with an endodontic instrument in the canal, master-cone radiographs, and postoperative radiographs, were reviewed collectively when determining procedural complications. All procedural complications were assessed on periapical radiographs according to predefined radiographic criteria adapted from previous studies [8,23].

### 2.4. Statistical Analysis

Complete agreement was observed among the three evaluators for all other procedural complications. The occurrence of any procedural complication was defined as the primary outcome, whereas analyses of individual complication types were considered secondary/exploratory outcomes.

Continuous variables were expressed as medians and interquartile ranges (IQR) based on the non-normal distribution verified by the Shapiro–Wilk test, while categorical variables were presented as frequencies and percentages.

Due to dependency arising from individual students performing multiple endodontic treatments (repeated measurements), mixed-effects regression modelling was used to account for the hierarchical structure and clustering within the dataset. For the primary outcome (presence of any procedural complication), a generalized linear mixed model (GLMM) with a logit link function was fitted, including training status, tooth type, the interaction between training status and tooth type, and the number of treated teeth per student as fixed effects. For the secondary exploratory analyses, separate GLMMs were fitted for each individual complication type, including training status and tooth type as fixed effects. In all models, the student was included as a random intercept to account for intra-student correlation. Odds ratios (ORs) with 95% confidence intervals (CIs) were calculated. Statistical significance was set at *p* < 0.05. All statistical computations were performed using R software (version 4.6.0; R Foundation for Statistical Computing, Vienna, Austria).

## 3. Results

Inter-examiner agreement was almost perfect for the assessment of root canal filling homogeneity (Fleiss’ κ = 0.994), while complete agreement was observed for all other procedural complications. A total of 202 dental students were enrolled in the study, consisting of 90 students in the non-training cohort and 112 students in the structured training/OSCE/OSPE group (Table 1). Throughout the study period, these students treated a combined total of 1204 teeth. The non-training group completed 722 endodontic treatments, whereas the training group completed 482 treatments. Regarding the anatomical distribution of the treated teeth, the non-training group performed treatments on 230 (31.9%) anterior teeth, 182 (25.2%) molars, 127 (17.6%) single-rooted premolars, and 183 (25.3%) two-rooted premolars. In comparison, the training group treated 135 (28%) anterior teeth, 111 (23%) molars, 127 (26.4%) single-rooted premolars, and 109 (22.6%) two-rooted premolars. The median number of treated teeth per student was higher in the non-training group (Median = 8, IQR: 7–9) than in the structured training/OSCE/OSPE group (Median = 4, IQR: 3–5). At least one procedural complication was identified in 310 of 722 treated teeth (42.9%) in the non-training cohort and in 182 of 482 teeth (37.8%) in the training cohort.

**Figure 1:**
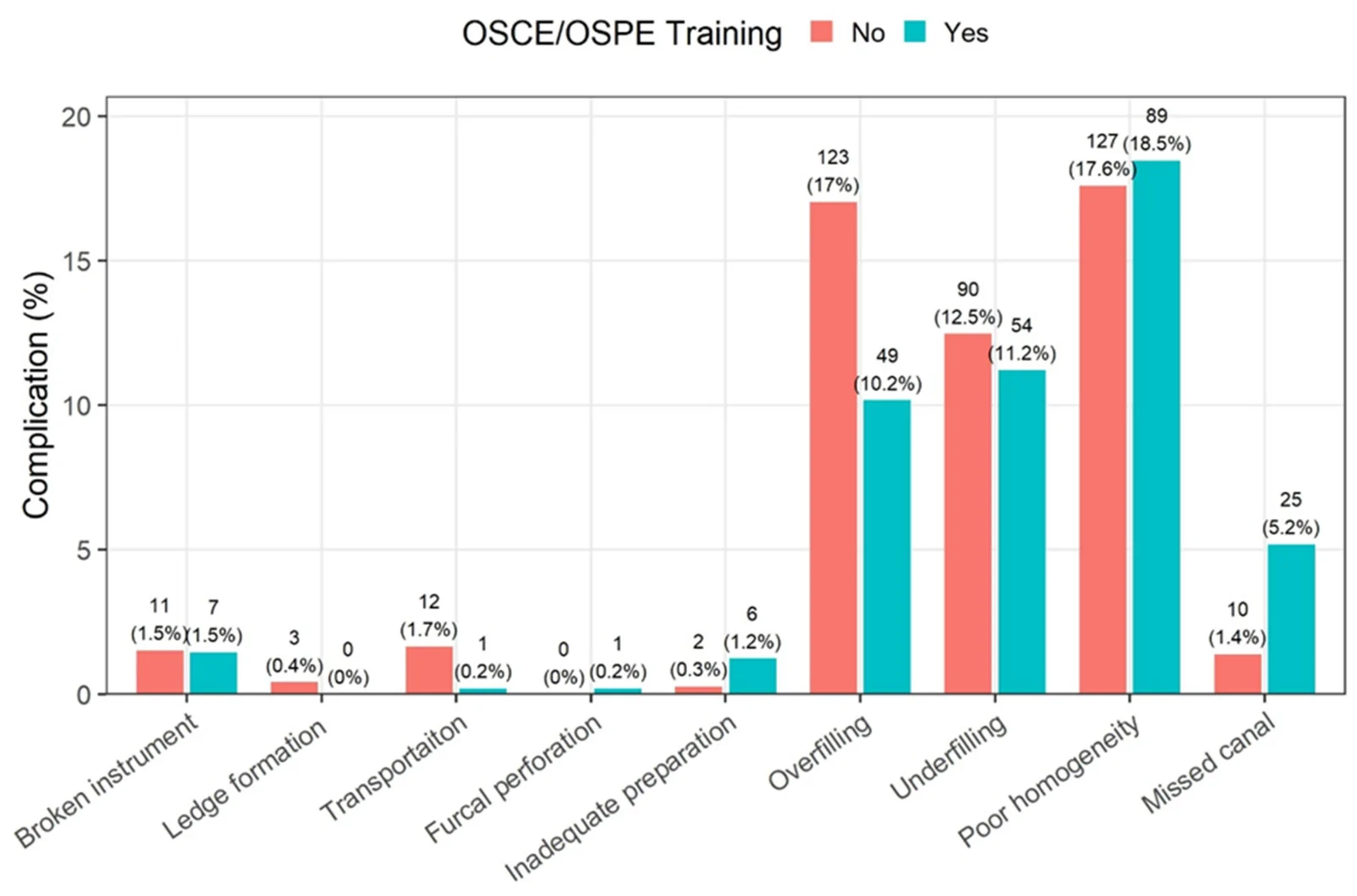
Frequency of procedural complications in all treated teeth according to training status.

The descriptive distribution of individual procedural complications across both student cohorts is illustrated in Figure 1. Among the recorded complications, poor homogeneity was the most frequent error encountered in both groups, affecting 17.6% (n = 127) of the non-training cohort and 18.5% (n = 89) of the structured training/OSCE/OSPE group. Overfilling was observed in 17.0% (n = 123) of teeth in the non-training group compared with 10.2% (n = 49) in the structured training/OSCE/OSPE group. Conversely, missed canals were more prevalent in the training group, occurring at a rate of 5.2% (n = 25) compared to 1.4% (n = 10) in the non-training group. Complications, such as broken instruments, ledge formation, and furcal perforations, consistently demonstrated minimal clinical prevalence (below 2%) across both educational cohorts. Lateral perforation was not observed in any of the groups.

The primary GLMM analysis presented in Table 2 revealed that OSCE/OSPE-based assessment and structured preclinical training were not significantly associated with the overall likelihood of procedural complications (OR = 0.58, 95% CI: 0.29–1.16, *p* = 0.069). The number of treated teeth per student was also not significantly associated with procedural complications (OR = 1.00, 95% CI: 0.90–1.12, *p* = 0.942). Among different tooth types, molar teeth were associated with a nearly six-fold increase in complication odds compared with anterior teeth (OR = 5.84, 95% CI: 3.66–9.46, *p* < 0.001). Conversely, single-rooted premolars (*p* = 0.889) and two-rooted premolars (*p* = 0.124) did not differ significantly from anterior teeth. Furthermore, no statistically significant interaction effects were observed between structured training/OSCE/OSPE group status and tooth types (*p* > 0.05), indicating that the association between training status and complication rates did not vary across different tooth types.

**Figure 2:**
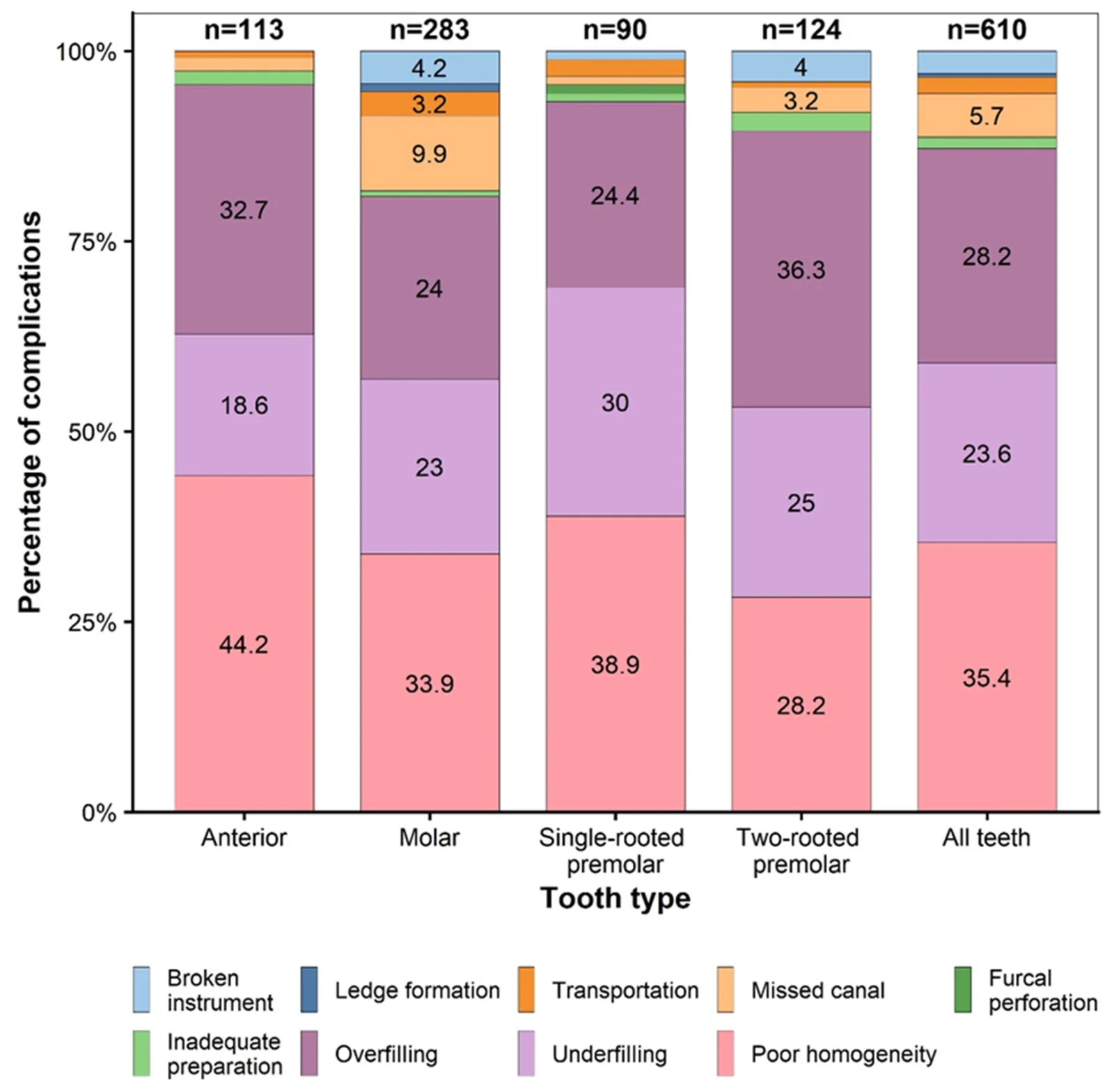
Types and prevalence (%) of procedural complications across tooth type. (For graphical clarity, numerical percentage labels below 3% are not displayed; however, all complication categories are represented in the figure).

The descriptive distribution of specific complication types across different tooth groups and all treated teeth is presented in Figure 2. Across all combined categories, poor homogeneity (35.4%), overfilling (28.2%), and underfilling (23.6%) represented the vast majority of procedural errors, while missed canals accounted for 5.7%. Tooth type influenced error patterns, particularly in molar teeth where poor homogeneity (33.9%), overfilling (24.0%), underfilling (23.0%), missed canals (9.9%), and broken instruments (4.2%) were observed. In contrast, anterior teeth were predominantly characterized by poor homogeneity (44.2%) and overfilling (32.7%), while showing a lower diversity of other errors. Single-rooted and two-rooted premolars followed a similar trend, dominated by filling-related errors (poor homogeneity, underfilling, and overfilling), which together comprised over 80% of the complications in these groups. Among all 610 recorded complication events, the less frequent categories were broken instrument (n = 18, 2.95%), transportation (n = 13, 2.13%), inadequate preparation (n = 8, 1.31%), ledge formation (n = 3, 0.49%), and furcal perforation (n = 1, 0.16%); no lateral perforation was observed.

As secondary exploratory analyses, separate GLMMs adjusted for tooth type were constructed to examine the associations between educational cohort and individual procedural complication types (Table 3). These analyses were performed without adjustment for multiple comparisons. In these exploratory analyses, lower odds of transportation (OR = 0.12, 95% CI: 0.02–0.94, *p* = 0.044) and overfilling (OR = 0.56, 95% CI: 0.38–0.82, *p* = 0.003) were observed in the training group. Conversely, the odds of missed canals (OR = 6.23, 95% CI: 2.08–18.65, *p* = 0.001) were significantly higher in the training cohort. No statistically significant differences were detected between the groups regarding inadequate preparation (OR = 4.80, 95% CI: 0.96–23.99, *p* = 0.056), underfilling (OR = 0.87, 95% CI: 0.56–1.35, *p* = 0.525) and poor homogeneity (OR = 1.11, 95% CI: 0.77–1.62, *p* = 0.568). Due to low clinical prevalence, reliable models could not be fitted for broken instrument, ledge formation, furcal perforation, and lateral perforation. Given the multiple secondary outcomes evaluated, these findings should be interpreted as exploratory.

## 4. Discussion

The current undergraduate curriculum guidelines of the European Society of Endodontology recommend that students demonstrate the necessary skills in a preclinical setting before performing endodontic treatment on patients and that their theoretical knowledge, practical skills, and clinical decision-making competencies be supported through formative and summative assessments [19]. In this context, training provided on simulated models in phantom laboratories and OSPE/OSCE-based assessment systems has emerged as an educational method that has become increasingly widespread in dental schools, particularly over the last decade [24].

Investigating the experiences of instructors and dental students regarding the educational and assessment approaches implemented in dental schools is of great importance for determining whether an adequate standard of treatment is being achieved [25]. In the present study, procedural complications observed during clinical root canal treatment were compared between a group that had received structured training in phantom laboratories and had undergone OSCE/OSPE-based assessment and a group that had not participated in this training and assessment. The overall complication rates were 42.9% and 37.8% in the groups without and with training, respectively. In the primary analysis, however, OSCE/OSPE-based assessment and structured preclinical training were not significantly associated with the overall occurrence of procedural complications (OR = 0.58, 95% CI: 0.29–1.16, *p* = 0.069). The confidence interval included 1.0, indicating that the observed data are compatible with both a modest protective association and no association. This finding is consistent with studies evaluating the effect of additional preclinical endodontic training on the quality of treatment in the clinical setting [6,19,26]. The absence of a dramatic change in the training group may be explained by Chambers’ definition of the “learning curve” between practice and competence, as also described by Barakat et al. [23,27]. This concept states that once a certain point has been reached, additional practice beyond that point no longer contributes to skill acquisition. Another study on the acquisition of medical clinical skills through practice also reported that the duration of practice plays a role in achieving the “initial mastery” of basic skills; however, subsequent improvement is achieved not through repetition but through the completion of competencies in deficient areas [28].

Previous studies have used a wide variety of criteria when classifying root canal fillings on radiographic images as acceptable or unacceptable. The procedural complications evaluated in the present study were selected from among the commonly assessed and reported complications used in the literature to determine the quality of root canal fillings [6,8,23].

When the procedural complications observed between the groups were compared, inadequate homogeneity was found to be the most frequently encountered complication in both groups, with rates of 17.6% in the group without training and 18.5% in the group with training. No significant difference was observed between the groups.

In the literature, when the homogeneity of root canal fillings has been evaluated, the rates of inadequate density/homogeneity in preclinical and clinical student studies have shown a wide range of variation. Some data in the studies reported acceptable density rates ranging from 6% to 34.2% [29,30]. One of the most common reasons for unacceptable root canal fillings is related to filling density and homogeneity problems. It has been reported that the low-density and non-homogeneous structure of the root canal filling increases the likelihood of adversely affecting the treatment outcome and that homogeneous root canal fillings without voids are associated with better clinical outcomes [31,32].

In the literature, studies conducted on students have reported apical transportation and overfilling or underfilling of the root canal as frequent and significant procedural errors [33,34,35].

Unlike the rates reported in the literature, in the present study, the rate of overfilling was observed to be higher than that of underfilling [36,37]. This finding was interpreted as indicating that the enlargement performed by the students to avoid leaving uncleaned areas at the root apex resulted in the loss of working length.

In the secondary/exploratory analyses, exposure to the revised educational program was associated with lower odds of canal transportation and overfilling compared with the traditional education cohort. The odds of underfilling were also numerically lower; however, this association was not statistically significant. Although these findings may be related to differences in the educational programs, the retrospective comparison of two consecutive academic cohorts does not allow the observed associations to be attributed specifically or causally to phantom laboratory training or OSCE/OSPE-based assessment. Similar to the findings obtained in the present study, it has been reported that student groups receiving additional training achieved more appropriate root canal filling length and exhibited fewer procedural errors [23,38]. Better root canal filling length and overall root canal filling performance have also been reported in groups receiving structured instruction [39]. Similar improvements have also been observed in revised teaching and supervision programs. In participants enrolled in the new program, the rate of high-quality root canal fillings increased to as high as 62% [11].

In contrast, missed canals were more common in the training group, with a rate of 5.2%, whereas this rate was 1.4% in the non-training group. In the literature, similar to the findings of the present study, this rate is generally low in student clinics and ranges from approximately 0.12% to 5.6% [40,41,42]. This finding should be interpreted cautiously, as differences in tooth anatomy, anatomical complexity, and case difficulty between the cohorts cannot be excluded. Although efforts were made to assign cases with comparable levels of difficulty, baseline case complexity was not objectively quantified. In addition, the retrospective design did not allow detailed standardization of anatomical characteristics between the two cohorts. Therefore, the higher odds of missed canals cannot be attributed to shortcomings in the educational intervention and may have been influenced by differences in case mix or other unmeasured confounding factors. Although reliance on standardized simulated tooth geometries could theoretically limit exposure to natural anatomical variability, this explanation is unlikely to fully account for the present finding because the phantom laboratory training also included extracted natural teeth embedded in simulated clinical models. Nevertheless, the present study was not designed to determine the mechanism underlying the higher occurrence of missed canals, and this unexpected finding should therefore not be attributed to any specific component of the educational program.

Procedural complications such as broken instruments, ledge formation, and perforation were infrequent in both educational groups. No lateral perforation was observed in either group. The data were found to be consistent with studies in the literature evaluating procedural complications among dental students. El-Ma’aita et al. [42] reported ledge formation in 2.8%, perforation in 1.4%, and instrument separation in 3.8% of cases. Alghamdi et al. [43] reported ledge formation in 3.0%, apical perforation in 1.0%, and coronal perforation in 0.5% of cases. Dadresanfar et al. [44] reported that no perforation was observed. These findings suggest that differences between the educational cohorts may be more apparent for specific technical outcomes than for the overall occurrence of procedural complications.

Tooth type was one of the factors most strongly associated with the development of procedural complications in the present study, and the likelihood of procedural complications was approximately six times higher in molars than in anterior teeth. This finding supports previous endodontic studies conducted on undergraduate students. Studies evaluating root canal treatments performed by undergraduate clinical students have reported that the rates of procedural errors in molars are higher than those in anterior and premolar teeth [41,45]. The more complex root canal anatomy of molars, the presence of a greater number of root canals, limited visibility and access in the posterior region, and the fact that working length determination, canal preparation, and obturation require a higher level of technical skill may explain this difference. Current studies also show that molar root canal treatment continues to be an important area of technical training for undergraduate dental students.

Furthermore, no statistically significant interaction effect was observed between educational status and tooth type in the present study. This indicates that the association between training status and procedural complication rates did not differ according to tooth type. In the literature, direct evidence regarding the interaction between education and tooth type is limited. Studies have reported associations of education and tooth type with procedural complications; however, their interaction has not been tested [46]. The primary null hypothesis of the present study was not rejected.

Several limitations should be considered. First, this was a retrospective, single-center study comparing two consecutive academic cohorts, and therefore unmeasured cohort- and time-related differences cannot be excluded. Potential differences in student characteristics, clinical supervision, and other unmeasured temporal factors between the two academic years may therefore have influenced the observed outcomes. Although both cohorts participated in clinical observation and the endodontic observation component lasted two weeks in each cohort, the overall duration and structure of the Clinical Observation course differed between academic years. Moreover, phantom laboratory training and scenario-based OSCE/OSPE assessment were introduced as components of the revised educational program in the 2024–2025 cohort. Because these educational modifications occurred concurrently, the independent contribution of each component cannot be determined.

The number of treatments performed per student differed substantially between the groups, which may have introduced the potential confounding effect of clinical treatment volume. Although efforts were made to assign patients with comparable levels of difficulty, complete standardization of case complexity could not be guaranteed. The assessment of procedural complications was based on two-dimensional periapical radiographs, which have inherent limitations in detecting certain procedural complications, particularly canal transportation, ledge formation, perforation, and missed canals. Although available radiographs obtained at different stages of treatment were reviewed collectively, the two-dimensional nature of these images may have limited the detection of subtle or superimposed anatomical and procedural findings. In addition, the low prevalence of rare procedural complications, including instrument separation, ledge formation, and lateral/furcal perforation, limited the statistical power to detect between-group differences and precluded reliable model estimation for these outcomes. Finally, the individual complication analyses were considered secondary/exploratory outcomes and were performed without adjustment for multiple comparisons. Therefore, the possibility of an increased risk of Type I error should be considered when interpreting these findings.

Furthermore, the specific structure, duration, and implementation of the educational program evaluated in this study may differ from phantom laboratory training and OSCE/OSPE-based assessment programs implemented at other dental schools; therefore, the findings may not be directly generalizable to other undergraduate endodontic curricula. Nevertheless, the evaluation of a large number of clinical treatments and the consideration of the dependency arising from multiple treatments performed by the same student using mixed-effects models are important strengths of the present study. In the future, similar studies involving larger student groups across different dental schools may be planned.

## 5. Conclusions

In conclusion, although the overall likelihood of procedural complications was lower among students who were exposed to structured preclinical phantom laboratory training and OSCE/OSPE-based assessment, this association did not reach statistical significance. However, the training was associated with a lower occurrence of certain procedural errors, particularly overfilling and canal transportation. Molar teeth were associated with a higher likelihood of procedural complications compared with anterior teeth. The higher frequency of missed canals in the training group should be interpreted cautiously, as differences in case selection, anatomical complexity, and other unmeasured confounding factors cannot be excluded.

The findings suggest that structured preclinical phantom laboratory training and OSCE/OSPE-based assessment may be valuable components of undergraduate endodontic education, although clinical performance is likely influenced by multiple educational, clinical, and case-related factors.

## Figures and Tables

**Table 1:** Distribution of treated teeth and endodontic complications among students with and without structured training and Objective Structured Clinical Examination (OSCE)/Objective Structured Practical Examination (OSPE).

	OSCE/OSPE and Training		
Variables	No	Yes	Total
Number of students	90	112	202
Number of treated tooth	722	482	1204
Tooth Type			
Anterior	230 (31.9%)	135 (28%)	
Molar	182 (25.2%)	111 (23%)	
Single-rooted premolar	127 (17.6%)	127 (26.4%)	
Two-rooted premolar	183 (25.3%)	109 (22.6%)	
Treated tooth per student (Median, IQR)	8 (7–9)	4 (3–5)	
Teeth with ≥1 procedural complication	310 (42.9%)	182 (37.8%)	

**Table 2:** Generalized Linear Mixed Model (GLMM) analysis evaluating the associations of structured training/OSCE/OSPE, tooth type, number of treated teeth per student, and the training × tooth type interaction with the likelihood of any procedural complication.

Variable	OR	95% CI	*p*
OSCE/OSPE and training (ref: No)	0.58	0.29–1.16	0.069
Treated tooth per student	1.000	0.90–1.12	0.942
Tooth Type (ref:Anterior)			
Molar	5.84	3.66–9.46	**<0.001**
Single-rooted premolar	1.04	0.62–1.73	0.889
Two-rooted premolar	1.42	0.91–2.23	0.124
Training × Tooth Type Interaction			
Training (Yes): Molar	1.62	0.74–3.53	0.228
Training (Yes): Single-rooted premolar	1.55	0.70–3.44	0.276
Training (Yes): Two-rooted premolar	1.55	0.72–3.32	0.260

OR = Odds Ratio; CI = Confidence Interval. Statistically significant *p*-values (*p* < 0.05) are indicated in bold. For the main effects, no training and anterior teeth served as the reference categories, respectively. For the interaction effects, training (yes) and anterior teeth were utilized as the baseline references.

**Table 3:** GLMM analysis of individual procedural complications according to structured training/OSCE/OSPE group status.

Outcome (Complications)	OR	95% CI	*p*
Transportation	0.12	0.02–0.94	**0.044**
Inadequate preparation	4.80	0.96–23.99	0.056
Overfilling	0.56	0.38–0.82	**0.003**
Underfilling	0.87	0.56–1.35	0.525
Poor homogeneity	1.11	0.77–1.62	0.568
Missed canal	6.23	2.08–18.65	**0.001**

OR = Odds Ratio; CI = Confidence Interval. Statistically significant *p*-values (*p* < 0.05) are indicated in bold. Models are adjusted for tooth type. No training was defined as the reference category for each model. Models for broken instrument, ledge formation, furcal perforation and lateral perforation were not fitted due to low prevalence.

## Data Availability

The data supporting the findings of this study are available from the corresponding author upon reasonable request. The data are not publicly available due to privacy and ethical restrictions involving patient information.

## Abbreviations

The following abbreviations are used in this manuscript:

OSCE	Objective Structured Clinical Examination
OSPE	Objective Structured Practical Examination
GLMMs	Generalized Linear Mixed Models
ESE	European Society of Endodontology
IQR	Interquartile Ranges
ORs	Odds Ratios
CIs	Confidence Intervals
